# Neurostructural correlates of retinal microvascular caliber in adolescent bipolar disorder

**DOI:** 10.1002/jcv2.12029

**Published:** 2021-08-22

**Authors:** Megan Mio, Kody G. Kennedy, Mikaela Dimick, Alysha Sultan, Lisa Fiksenbaum, Beth Selkirk, Peter Kertes, Brian W. McCrindle, Sandra E. Black, Bradley J. MacIntosh, Benjamin I. Goldstein

**Affiliations:** ^1^ Centre for Youth Bipolar Disorder Centre for Addiction and Mental Health Toronto Ontario Canada; ^2^ Department of Pharmacology and Toxicology University of Toronto Toronto Ontario Canada; ^3^ Sunnybrook Research Institute Sunnybrook Health Sciences Centre Toronto Ontario Canada; ^4^ Department of Ophthalmology and Vision Sciences John and Liz Tory Eye Centre Sunnybrook Health Sciences Centre Toronto Ontario Canada; ^5^ Department of Ophthalmology and Vision Sciences University of Toronto Toronto Ontario Canada; ^6^ Labatt Family Heart Centre Hospital for Sick Children Toronto Ontario Canada; ^7^ Department of Pediatrics Faculty of Medicine University of Toronto Toronto Ontario Canada; ^8^ Hurvitz Brain Sciences Research Program Sunnybrook Health Sciences Centre Toronto Ontario Canada; ^9^ Department of Medical Biophysics University of Toronto Toronto Ontario Canada; ^10^ Department of Psychiatry University of Toronto Toronto Ontario Canada

**Keywords:** adolescent, bipolar disorder, brain structure, retinal vessels

## Abstract

**Objectives:**

Vascular‐brain associations are well established in adults but neglected in youth and psychiatric populations, who are at greater cardiovascular risk. We therefore examined the association of retinal vascular caliber with regional brain structure in adolescents with and without bipolar disorder (BD).

**Methods:**

One hundred and three adolescents (*n* = 51 BD, *n* = 52 healthy control [HC]) completed retinal fundus imaging, yielding arteriolar and venular diameters, followed by T1‐weighted 3‐Tesla MRI. Region of interest (ROI) analyses examined ventrolateral prefrontal cortex (vlPFC) and ventromedial prefrontal cortex (vmPFC), anterior cingulate cortex (ACC), amygdala, and hippocampus, complemented by vertex‐wise analyses. Linear regression assessed the association between retinal measures and brain structure, adjusting for covariates including age, sex, BMI, and intracranial volume (ICV).

**Results:**

In the overall sample, arteriolar caliber was negatively associated with ACC volume (*β* = −0.20, *p*_uncorrected_ = .046) and surface area (*β* = −0.19, *p*_uncorrected_ = .049). There were no other significant ROI findings. Vertex‐wise analyses detected several significant positive bilateral associations of arteriovenous ratio (AVR) with volume and surface area in regions including rostral middle frontal gyrus (left *p* = .001; right *p* = .006), isthmus cingulate cortex (left and right *p* < .001), and left precuneus (*p* < .001). Significant negative associations were also observed for AVR (*p* = .03) and arteriolar caliber (*p* = .01), including a cluster encompassing the left rostral middle frontal gyrus and orbitofrontal cortical thickness. In the sole retinal‐by‐diagnosis interaction, greater AVR was more strongly associated with lower volume in the left middle temporal and fusiform gyri in BD versus HC (*p* = .004).

**Conclusion:**

This study provides evidence that vascular‐brain associations are already evident in adolescence, suggesting that optimizing cardiovascular health may benefit the brain. This may be particularly relevant in BD and other brain disorders. Future research focusing on subpopulations where vascular‐brain associations may be especially strong, for whom vascular‐related interventions may be most indicated, is warranted.

**Key points**

- Anomalous retinal vascular caliber has been observed in bipolar disorder (BD). Studies in non‐psychiatric populations suggest that retinal vessels, well‐established proxies for cerebral microvessels, are related to brain structure and function.
- Our results show that retinal vascular caliber is significantly associated with cortical thickness, volume, and surface area in mood‐related brain regions, including the cingulate, the rostral middle frontal gyrus, and orbitofrontal cortex in both BD and healthy control adolescents.
- Optimizing cardiovascular health may offer direct brain benefits, particularly in the context of psychiatric disorders. Future research should focus on subpopulations where vascular‐brain associations may be particularly strong, for whom vascular‐targeted interventions may be most beneficial.

## INTRODUCTION

Bipolar disorder (BD) is a disabling psychiatric condition affecting between 2% and 5% of adolescents (Kozloff et al., 2010; Van Meter et al., 2019). In addition to psychiatric symptoms and cognitive impairment, BD confers elevated risk of early‐onset cardiovascular disease (CVD), a risk that cannot be explained by traditional cardiovascular risk factors (CVRFs) alone (Goldstein, 2017; Goldstein et al., 2020). Evidence suggests that BD and CVD share common underlying pathophysiology and that the link between BD and cardiovascular dysfunction likely begins as early as adolescence (Goldstein, 2017; Goldstein et al., 2015). Examining cerebrovascular phenotypes may inform understanding of the BD‐CVD link.

Imaging of retinal microvessels has emerged as a promising window into the vascular‐brain link. Retinal microvessels can be easily, affordably, and noninvasively visualized using standard ophthalmic cameras (Heringa et al., 2013; Meier et al., 2013). Retinal arterioles and venules are the best available proxy of their cerebral microvascular counterparts as they are anatomically homologous and share common embryologic origins and physiologic autoregulatory mechanisms (Kwa, 2006; Patton et al., 2005; Shalev et al., 2013; Wong et al., 2001). Importantly, retinal vessels are a sensitive and robust measure of vascular health. Retinal vascular caliber is associated with CVRFs (Liew et al., 2008; Ogagarue et al., 2013; Wong et al., 2006) and can serve as an indicator of risk for future stroke (Ikram et al., 2006), vascular dementia, and cerebral small vessel disease (Cheung, et al, 2014; Heringa et al., 2013). Cardiovascular risk factors including elevated BMI and blood pressure are associated with retinal vascular caliber in children and adolescents (Li et al., 2016; Zheng et al., 2013).

Retinal fundus photography yields three parameters: the central retinal arteriolar equivalent (CRAE), central retinal venular equivalent (CRVE), and arteriovenous ratio (AVR), a ratio of CRAE:CRVE. These measures are commonly calculated using a standard (Hubbard et al., 1999; Knudtson et al., 2003). Lower AVR is generally indicative of elevated cardiovascular risk (Wong et al., 2006). Similarly, lower CRAE and higher CRVE are suspected to indicate higher cardiovascular risk, although there is mixed evidence (Naiberg et al., 2017; Sun et al., 2009; Wong et al., 2006). In the Multi‐Ethnic Study of Atherosclerosis (MESA) sample, lower arteriolar caliber was more strongly associated with systolic blood pressure and hypertension status, while larger venular caliber was associated with diabetes, serum glucose, and plasma triglycerides (Wong et al., 2006). Relative to healthy controls (HCs), those with BD and schizophrenia display anomalous retinal vascular caliber (Appaji et al., 2019; Meier et al., 2013). In one study, adults with BD had significantly lower retinal arteriolar caliber as compared to those with schizophrenia (Appaji et al., 2019). In adolescents with BD, AVR is negatively associated with mood symptom severity and positively correlated with a peripheral arterial tonometry measure of endothelial function (Naiberg et al., 2017). Furthermore, in a large population‐based study of 865 adolescents and young adults, symptoms of depression and anxiety were associated with wider retinal arteriolar caliber, even after controlling for CVRFs (Meier et al., 2014).

Despite documented links between retinal vascular caliber, psychiatric symptoms, and neurocognition (Heringa et al., 2013), few studies have evaluated the link between retinal vascular caliber and gray matter neurostructural phenotypes, and none have looked at this in any psychiatric population of any age group. The important relationship between cardiovascular and brain health has long been acknowledged in a number of adult and nonpsychiatric populations including vascular dementia and stroke (Girouard & Iadecola, 2006; Pase et al., 2018; Seo et al., 2010). CVRFs are associated with brain structure in a number of populations, including BD (Hajek et al., 2014; Islam et al., 2017; Kennedy et al., 2021). While the literature examining vascular‐brain associations in youth is limited, anomalous neuroimaging findings have been reported in youth at cardiovascular risk. In a study of obese adolescents with metabolic syndrome (MetS) (Yau et al., 2014), the MetS adolescents had significantly lower retinal arteriolar caliber relative to HCs, and lower retinal arteriolar caliber was associated with subclinical white matter microstructural damage.

Taken together, retinal microvessels, which comprise proxies for cerebral microvessels, may be relevant to brain structure in BD. We previously found that the association of CVRFs with brain structure and function is stronger among adolescents with BD versus HC (Islam et al., 2017; Kennedy et al., 2021; Naiberg et al., 2017). We therefore hypothesized that the association between retinal vascular caliber and brain structure will also differ between adolescents with BD and HC. We examined regions of interest (ROIs) that are known to be relevant to BD and/or cerebrovascular risk: the anterior cingulate cortex (ACC), ventrolateral prefrontal cortex (vlPFC), ventromedial prefrontal cortex (vmPFC), hippocampus, and amygdala. Given the lack of prior studies, we did not hypothesize a particular direction of association.

## METHODS

### Participants

Participants were 103 adolescents, 51 BD, and 52 HC. Participants meeting criteria for either BD‐I, BD‐II, or BD‐not otherwise specified (NOS) were recruited from the Centre for Youth Bipolar Disorder, a tertiary subspecialty clinic. Community advertisements were used to recruit healthy control participants.

Exclusion criteria were: unable to provide informed consent (e.g., severe psychosis and developmental delay), neurological or psychological impairment, existing cardiac or inflammatory conditions, infectious illness within the past 14 days, substance dependence within the past 3 months, currently taking anti‐inflammatory, anti‐lipidemic, or anti‐hypertensive agents, and those with contraindications to MRI. Additionally, HC participants were excluded if they had any lifetime major psychiatric diagnosis (i.e., BD, schizophrenia, major depressive disorder, and psychosis), met criteria for an anxiety disorder or alcohol or substance dependence in the past 3 months, or had a family history (in any first‐ or second‐degree relative) of BD or psychotic illness. Prior to any procedures, written informed consent was obtained from both participants and a parent or guardian. The research ethics board at Sunnybrook Health Sciences Centre approved all procedures.

### Diagnostic interviews

Psychiatric diagnoses were confirmed using the Schedule for Affective Disorders and Schizophrenia for School Age Children, Present and Lifetime version (K‐SADS‐PL) (Kaufman et al., 1997). The K‐SADS‐PL is a validated, semi‐structured interview used to assess psychiatric diagnoses in children and adolescents aged 7–18 years old in accordance with *Diagnostic and Statistical Manual of Mental Disorders, 4th Edition (DSM‐IV)* criteria. Participants were enrolled from 2014 to 2019. Mania and depression symptoms were scored through the K‐SADS Mania Rating Scale (Axelson et al., 2003) and K‐SADS Depression Rating Scale (Chambers et al., 1985), respectively. Current mood state was determined by symptom scores during the worst week in the month preceding the MRI scan. Additional metrics used to assess sociodemographic variables in Table 1 can be found in the Supporting Information.

**TABLE 1:** Demographic and clinical characteristics

Group (mean ± SD or %)					
Demographics	BD (*n* = 51)	HC (*n* = 52)	Statistical test	Effect size	*p*‐value
Age (years)	17.70 ± 1.76	17.34 ± 1.65	*t* = −1.07	*d* = 0.21	.29
Female (%)	33 (65)	24 (46)	*χ* ^2^ = 3.59	*V* = 0.19	.06
Race (Caucasian %)	35 (69)	26 (50)	*χ* ^2^ = 3.70	*V* = 0.19	.05
Intact family	32 (63)	38 (73)	*χ* ^2^ = 1.26	*V* = 0.11	.26
Socioeconomic status	4.20 ± 0.98	4.52 ± 0.58	*U* = 1133.0	*d* = 0.40	.04
Age of onset (years)	15.29 ± 1.92	‐	‐	‐	‐
BD subtype					
BD‐I	23 (45)	‐	‐	‐	‐
BD‐II	15 (29)	‐	‐	‐	‐
BD‐NOS	13 (25)	‐	‐	‐	‐
**Clinical characteristics**					
Lifetime psychosis	16 (31)	‐	‐	‐	‐
Lifetime SA	9 (18)	‐	‐	‐	‐
Lifetime SIB	28 (55)	1 (2)	*χ* ^2^ = 25.16	*V* = 0.54	<.001*
Lifetime SI	34 (66)	4 (8)	*χ* ^2^ = 25.68	*V* = 0.55	<.001*
Legal history (Police contact/arrest)	7 (14)	‐	‐	‐	‐
Lifetime physical abuse	1 (2)	‐	‐	‐	‐
Lifetime sexual abuse	4 (8)	‐	‐	‐	‐
Lifetime any abuse	5 (10)	‐	‐	‐	‐
Lifetime psychiatric hospitalization	24 (47)	‐	‐	‐	‐
Depression score—Current	15.02 ± 11.61	0.55 ± 1.79	*U* = 123.0	*d* = 2.58	.001*
Depression score—Most severe past	31.14 ± 11.11	3.61 ± 6.12	*U* = 66.50	*d* = 1.67	<.001*
Mania score—Current	6.43 ± 9.59	0.10 ± 0.40	*U* = 351.5	*d* = 0.93	<.001*
Mania score—Most severe past episode	33.94 ± 9.39	0.57 ± 1.76	*U* = 0.00	*d* = 1.85	<0.001*
CGAS—Most severe past episode	43.25 ± 9.73	‐	‐	‐	‐
CGAS—Highest level of functioning	72.75 ± 9.13	88.65 ± 5.73	*U* = 194.5	*d* = 1.45	<.001*
CGAS—Current episode (past month)	68.02 ± 12.35	88.52 ± 6.89	*U* = 150.5	*d* = 1.43	<.001*
**Lifetime comorbid diagnoses**					
ADHD	23 (45)	5 (10)	*χ* ^2^ = 16.38	*V* = 0.40	<.001*
ODD	15 (29)	‐	‐	‐	‐
Conduct disorder	1 (2)	‐	‐	‐	‐
Any anxiety	46 (90)	7 (14)	*χ* ^2^ = 60.69	*V* = 0.77	<.001*
SUD	11 (22)	‐	‐	‐	‐
**Family history (first or second degree)**					
Mania/hypomania	26 (51)	‐	‐	‐	‐
Depression	40 (78)	12 (23)	*χ* ^2^ = 34.63	*V* = 0.59	<.001*
Suicide attempts	5 (10)	‐	‐	‐	‐
Anxiety	35 (69)	8 (16)	*χ* ^2^ = 32.41	*V* = 0.57	<.001*
ADHD	15 (29)	2 (4)	*χ* ^2^ = 12.91	*V* = 0.36	<.001*
**Medication use—** **c** **urrent**					
SGA	31 (61)	‐	‐	‐	‐
Lithium	14 (27)	‐	‐	‐	‐
SSRI antidepressants	8 (16)	2 (4)	*χ* ^2^ = 4.12	*V* = 0.20	.04*
Non‐SSRI antidepressants	2 (4)	‐	‐	‐	‐
Stimulants	4 (7.8)	‐	‐	‐	‐
Valproate	0 (0)	‐	‐	‐	‐
Lamotrigine	8 (16)	‐	‐	‐	‐
**Medication use—lifetime**					
SGA	42 (82)	‐	‐	‐	‐
Lithium	16 (31)	‐	‐	‐	‐
SSRI antidepressants	25 (49)	3 (6)	*χ* ^2^ = 24.33	*V* = 0.49	<.001*
Non‐SSRI antidepressants	9 (18)	‐	‐	‐	‐
Stimulants	12 (24)	2 (4)	*χ* ^2^ = 8.49	*V* = 0.29	.004*
Valproate	4 (8)	‐	‐	‐	‐
Lamotrigine	13 (25)	‐	‐	‐	‐
Any medications	48 (94)	5 (10)	*χ* ^2^ = 73.60	*V* = 0.84	<.001*
**Physiological characteristics**					
Waist Circumference	80.47 ± 11.84	77.18 ± 11.87	*U* = 1123.0	*d* = 0.28	.16
Adjusted BMI	23.75 ± 4.59	22.54 ± 3.95	*U* = 570	*d* = 0.28	.23
Resting SBP	106.96 ± 10.74	109.08 ± 11.28	*t* = 0.97	*d* = 0.19	.33
Resting DBP	68.41 ± 6.77	67.80 ± 7.04	*t* = −0.45	*d* = 0.09	.66
Lifetime smoking	20 (39)	9 (17)	*χ* ^2^ = 6.11	*V* = 0.24	.01*

Abbreviations: ADHD, Attention‐Deficit Hyperactivity Disorder; BD, bipolar disorder; BMI, Body Mass Index; CE, current episode; CGAS, Children's Global Assessment Scale; DBP, Diastolic Blood Pressure; HC, healthy control; HP, highest in the past year; MSP, most severe past episode; ODD, Oppositional Defiant Disorder; SA, suicide attempt; SD, standard deviation; SGA, Second‐Generation Antipsychotic; SI, suicidal ideation; SIB, self‐injurious behavior; SUD, Substance Use Disorder; *n*, number of participants; SBP, Systolic Blood Pressure; SSRI, Selective Serotonin Reuptake Inhibitor.

*Significant group difference (*p* < .05).

### Retinal photography

Macula‐centered retinal fundus images were collected at a 50° angle using a Topcon 50 DX, Type 1A camera following pupil dilation with 1% tropicamide and 2.5% phenylephrine eye drops. Participants were asked to refrain from consuming any products containing caffeine or nicotine (cigarettes and electronic cigarettes). A Certified Ophthalmic Assistant (Beth Selkirk) performed all imaging. ImageJ Software (National Institutes of Health) was used to compute vessel diameters. Vessel diameters were converted from pixels to microns using a calibration coefficient of 4.92, and the Parr Hubbard Formula (Hubbard et al., 1999; Knudtson et al., 2003) was used to calculate CRAE and CRVE. AVR was computed as a ratio based on these values.

### Magnetic resonance imaging acquisition and processing

MRI data for this study were compiled from two imaging studies on two different scanners; as below, analyses controlled for scanner type. Gray and white matter were quantified in 3D T1‐weighted images, obtained using high‐resolution fast‐field echo imaging. Technical MRI sequences and a description of the FreeSurfer version 6.0 structural analysis pipeline can be found in the Supporting Information. This pipeline is described in greater detail in prior publications (Islam et al., 2017). Region of interest volumes were created by adding individual gyral labels from the Desikan‐Killiany atlas. The following ROIs were examined: (i) anterior cingulate cortex (ACC), (ii) ventrolateral prefrontal cortex (vlPFC), (iii) ventromedial prefrontal cortex (vmPFC), (iv) hippocampus, and (v) amygdala.

### Statistical analysis

All statistical analyses were performed in SPSS software version 25 (IBM Inc.). Independent‐sample *t*‐tests or Mann–Whitney *U* tests were used to determine group differences in continuous variables and *χ* ^2^ tests for categorical variables. Normality was confirmed using the Shapiro–Wilks test for continuous variables, and Levene's test for subsequent analysis of covariance (ANCOVA) analyses. ANCOVA analyses examined for between‐group differences in retinal measures controlling for age, sex, and lifetime smoking. Linear regression was used to examine associations between retinal measures and ROI structure, covarying for age, sex, BMI, and scanner (i.e., Siemens vs. Phillips). ICV was also included as a covariate when examining volume and surface area ROIs. Multiple testing was corrected on a family‐wise basis (dividing the significance level by the number of preselected ROIs, i.e., *α* = .05/5 = .01). For the vertex‐wise analysis, a general linear model examined the effect of retinal measures on brain structure, including the same covariates as our ROI approach. A vertex‐wise threshold of *p* < .05 was employed. To correct for multiple comparisons, Monte Carlo simulations thresholded at 1.3 (*p* < .05) were employed.

## RESULTS

### Demographic and clinical characteristics

This study included 103 adolescents, 51 BD (17.70 ± 1.76 years; 65% females), and 52 HC (17.34 ± 1.65 years; 46% females). The BD group had significantly lower socioeconomic status (*p* = .04) and had more female and Caucasian participants; however, these differences were nonsignificant (*p* = .06, *p* = .054, respectively). Lifetime history of tobacco smoking was significantly higher in the BD group (39% vs. 17%, *p* = .01). Psychiatric comorbidities and additional clinical characteristics are listed in Table 1.

### Retinal measures by group

Group differences in retinal measures are presented in Table 2. In univariate analyses, CRAE was significantly higher in the BD group (BD = 133.56 ± 24.99 μm, HC = 123.96 ± 18.42 μm); however, no significant difference was observed in CRVE or AVR. After controlling for age, sex, and tobacco smoking, CRAE remained significantly higher in the BD group than HC, *F* (1,101) = 4.70, *p* = .03, $\eta_{p}^{2}$ = 0.05). No significant between‐group differences were observed in AVR or CRVE in multivariable analyses.

**TABLE 2:** Retinal measures by group

	Group (mean ± SD)				
Retinal measures	BD (*n* = 51)	HC (*n* = 52)	Statistical test	Effect size	*p*‐value
AVR	0.64 ± 0.08	0.61 ± 0.08	*t* = −1.72	*d* = 0.34	.09
CRAE (μm)	133.56 ± 24.99	123.97 ± 18.42	*t* = −2.22	*d* = 0.44	.03*
CRVE (μm)	210.73 ± 37.42	204.68 ± 32.31	*t* = −0.88	*d* = 0.17	.38

Abbreviations: AVR, arteriovenous ratio; BD, bipolar disorder; CRAE, central retinal arteriolar equivalent; CRVE, central retinal venular equivalent; HC, healthy control.

*Significant group difference (*p* < .05).

### Region of interest analysis

**FIGURE 1:**
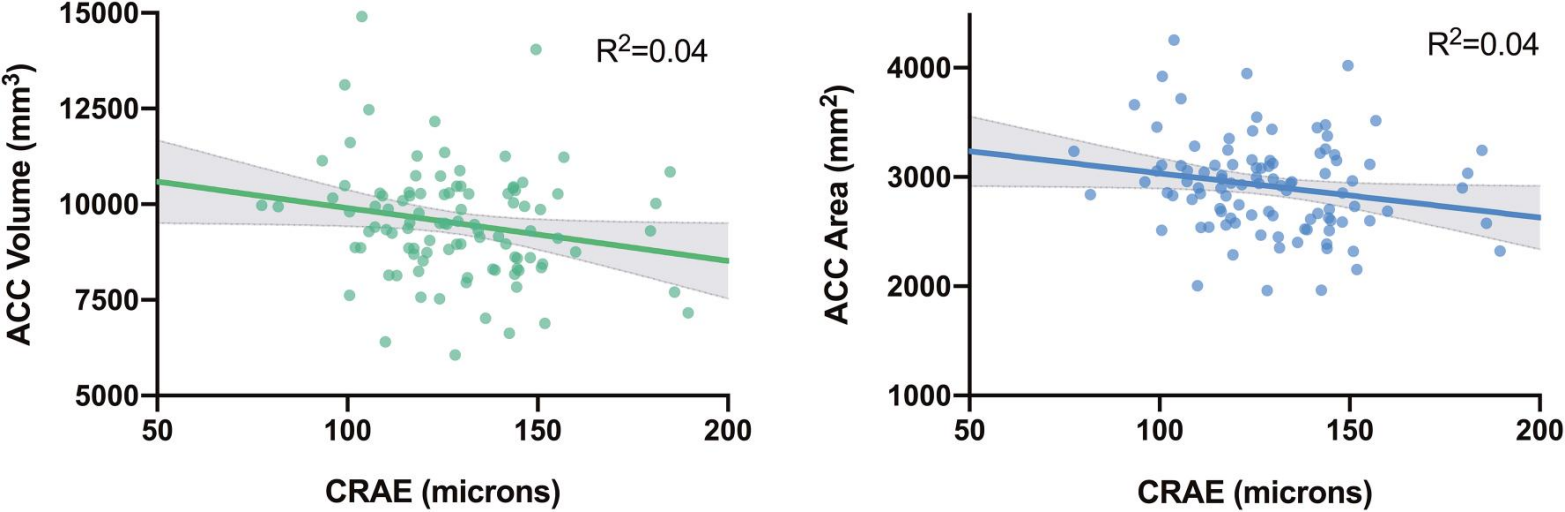
Association between central retinal arteriolar equivalent (CRAE) and regional brain structure in both bipolar disorder and healthy control groups, as measured through ROI approach. A significant main effect of CRAE was found on ACC volume (*β* = −0.2, *p*_uncorrected_ = .046) and ACC area (*β* = −0.19, *p*_uncorrected_ = .049), after correcting for age, sex, BMI, ICV, and scanner. ACC, anterior cingulate cortex; ICV, intracranial volume; ROI, region of interest

Associations of retinal measures on ROIs are presented in Table 3. There was a significant negative main effect of CRAE on ACC volume (*β* = −0.20, *p*_uncorrected_ = .046) and surface area (*β* = −0.19, *p*_uncorrected_ = .049) (see Figure 1). Neither of these findings were significant after correction for multiple comparisons (*α* = 0.01). Similarly, there was a significant diagnosis‐by‐CRAE interaction effect on ACC volume (*β* = 0.20, *p*_uncorrected_ = .049), whereby CRAE was associated with smaller ACC volume in BD versus HC. There were no significant findings for the other ROIs (see Table S1).

**TABLE 3:** Retinal main effect on brain structure

	ACC		vlPFC		vmPFC		Amygdala		Hippocampus	
	*β*	*p*	*β*	*p*	*β*	*p*	*β*	*p*	*β*	*p*
Volume										
AVR	−.13	.19	−.01	0.88	−.02	.82	−0.12	.11	−.07	.44
CRAE	−.20	.046*	−.14	0.14	.01	.86	−.08	.27	.02	.79
CRVE	−.11	.29	−.16	0.11	.02	.82	−.00	.99	.08	.37
Surface area										
AVR	−.11	.27	−.02	.79	−.03	.72				
CRAE	−.19	.049*	−.13	.18	−.04	.60				
CRVE	−.12	.22	−.13	.19	−.03	.73				
Cortical thickness										
AVR	.05	.60	−.01	.91	−.13	.21				
CRAE	.12	.22	−.07	.51	.06	.54				
CRVE	.08	0.41	−.08	.47	.17	.12				

Abbreviations: ACC, anterior cingulate cortex; AVR, arteriovenous ratio; CRAE, central retinal arteriolar equivalent; CRVE, central retinal venular equivalent; vlPFC, ventrolateral prefrontal cortex; vmPFC, ventromedial prefrontal cortex.

*Significant at *p* < .05 before multiple comparisons; **Significant at *p* < .05 after correction for multiple comparisons.

### Vertex‐wise analysis

There were a number of retinal main effects on brain structure in the whole sample (see Table S2), summarized below.

#### AVR findings

**FIGURE 2:**
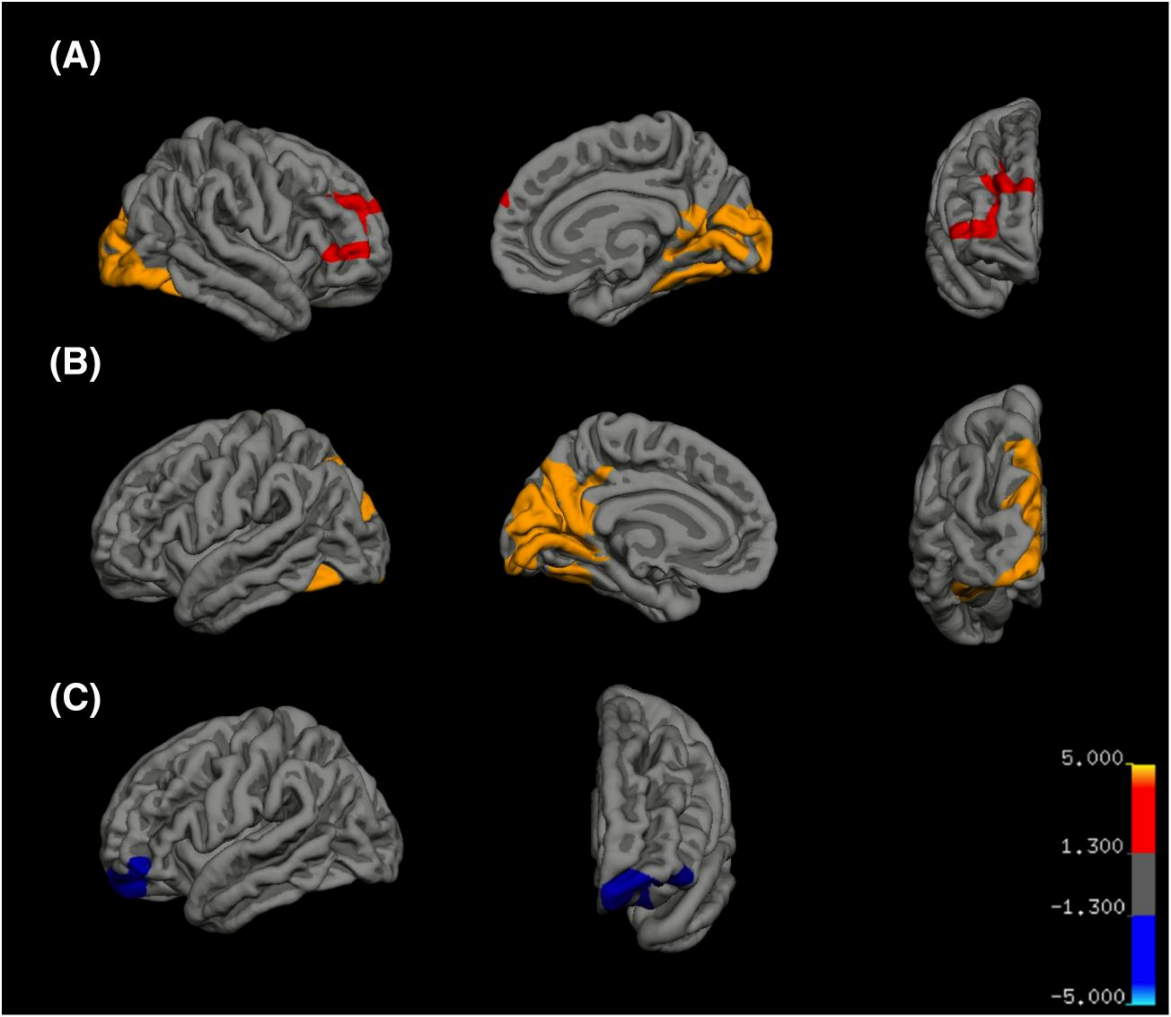
Log‐P map for corrected clusters of significant AVR main effect findings. (A) AVR main effect on rh isthmus cingulate volume (*p = *.0001) and rh rostral middle frontal volume (*p = *.006); (B) AVR main effect on lh isthmus cingulate volume (*p = *.0001); and (C) AVR main effect on lh rostral middle frontal thickness (*p = *.03). AVR, arteriovenous ratio

There was a significant positive association between AVR and cortical volume in three regionally distinct clusters (see Figure 2): right isthmus cingulate cortex, right rostral middle frontal gyrus, and left precuneus; lateral occipital cortex; and lingual and fusiform gyri. There was a significant negative association between AVR and cortical thickness in the left rostral middle frontal gyrus. Finally, there was a significant positive association between AVR and surface area in left isthmus cingulate cortex, right isthmus cingulate cortex, and right rostral middle frontal gyrus.

#### Arteriolar findings

There was a significant positive association between CRAE and cortical volume in the right lateral occipital cortex, right isthmus cingulate cortex, and left precuneus. There was a significant negative association between CRAE and cortical thickness in left supramarginal gyrus and left rostral middle frontal gyrus. Finally, there was a significant positive relationship between CRAE and surface area in the right rostral middle frontal gyrus, right lateral occipital cortex, and left isthmus cingulate cortex.

#### Venular findings

There was a significant positive association between CRVE and cortical volume in the right lateral occipital cortex, right isthmus cingulate cortex, right rostral middle frontal gyrus, and left precuneus. There was a significant negative association between CRAE and cortical thickness in the left supramarginal gyrus and left rostral middle frontal gyrus. Finally, CRVE was positively associated with surface area in the right lateral occipital, right rostral middle frontal gyrus, and left precuneus.

**FIGURE 3:**
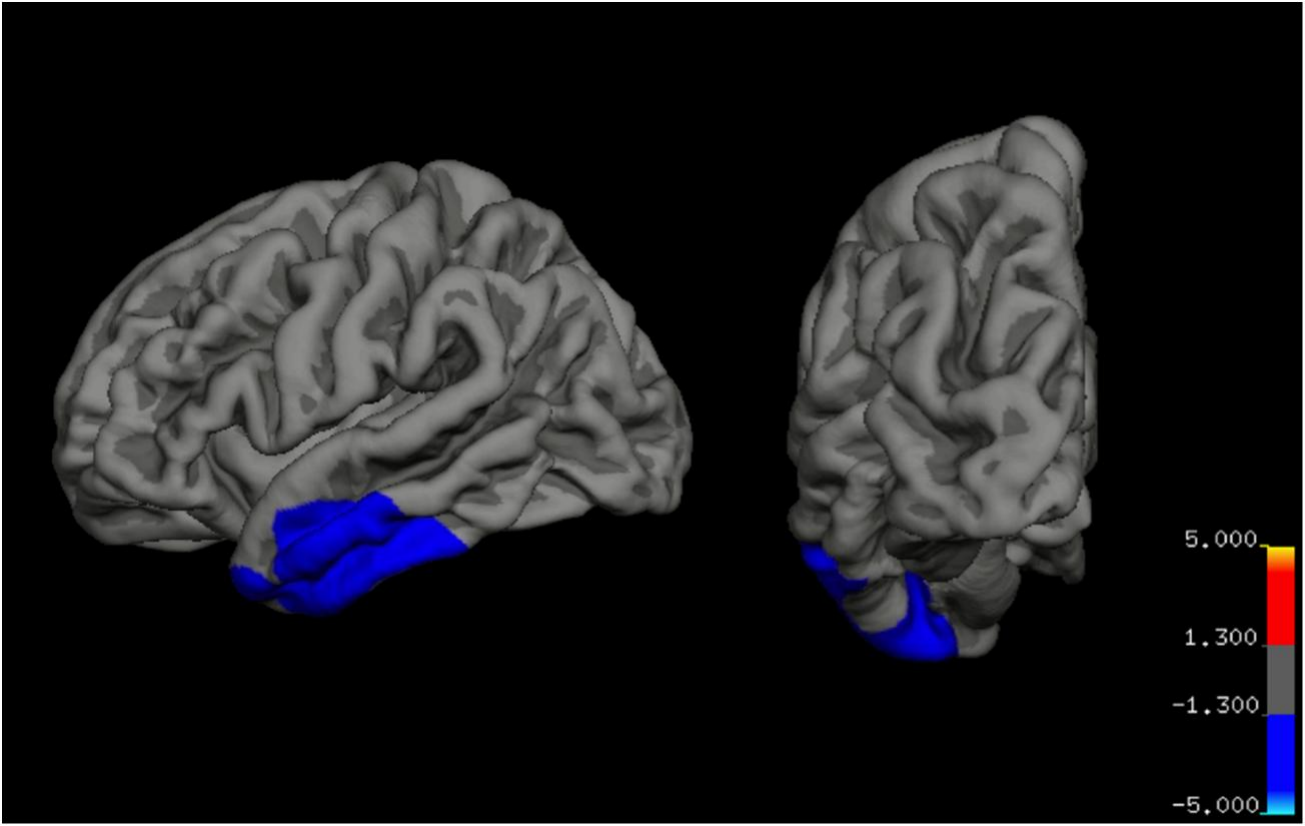
Log‐P map for corrected clusters of significant retinal‐by‐diagnosis interaction effects. AVR‐by‐diagnosis interaction effect on lh middle temporal gyrus volume (*p = *.004)

Significant retinal‐by‐diagnosis associations with brain structure, as measured through vertex‐wise analysis, are reported in Table S3. There was an interaction effect of AVR on surface area in the left middle temporal gyrus (overlap into the inferior temporal, superior temporal, and fusiform gyri; see Figure 3). In these regions, greater AVR was associated with lower cortical surface area to a significantly greater extent in BD versus HC. No other significant retinal‐by‐diagnosis interaction effects were detected for cortical thickness or volume or other retinal measurements. Vertex‐wise analyses were repeated after excluding all control participants with lifetime comorbid Attention‐Deficit Hyperactivity Disorder (ADHD) and/or anxiety (*n* = 5 ADHD, *n* = 7 anxiety). All vertex‐wise findings remained significant.

## DISCUSSION

We sought to investigate the relationship between retinal microvascular caliber—a proxy for cerebrovascular health—and brain structure among adolescents with and without BD. Present findings comprise proof‐of‐principle, insofar as retinal vascular caliber was associated with brain structure in this adolescent sample. Relative to adults, there is a dearth of studies on this topic in youth; we speculate that this may relate to assumptions about vascular‐brain associations only becoming relevant later in life. In the overall sample, vertex‐wise analyses detected a positive association between AVR and volume in the isthmus cingulate and rostral middle frontal gyrus. In ROI analyses, higher CRAE was associated with lower ACC volume and surface area in the overall sample. While there was limited evidence of diagnosis‐related interactions, regions significantly associated with retinal vascular caliber in this study are highly relevant to BD.

### Location and direction of findings

The cingulate cortex is a key brain region implicated in emotional regulation, cognition, and information processing (Strakowski et al., 2012). The importance of the ACC has been highlighted in studies of adult and youth with BD (Hibar et al., 2018; Phillips & Swartz, 2014; Toma, Islam, et al., 2019). Other regions identified in significant voxel‐wise clusters, including the rostral middle frontal gyrus, vlPFC, and lateral orbitofrontal cortex (OFC), are involved in emotion regulation, working memory, decision‐making, impulsivity, and reward function—all domains known to be impaired in BD (Beshkovet al., 2018; Chau et al., 2018; Lima et al., 2018; Rolls, 2019). Cortical thinning and volumetric reductions are well‐documented in these regions in both adolescent and adult BD (Hibar et al., 2018; Niu et al., 2017). Associations between retinal microvascular caliber and regional brain structure were evident across frontal, temporal, and occipital regions. Putative mechanisms underlying these regional differences are discussed below.

### Specific metrics

Findings regarding the rostral middle frontal gyrus exemplify the complexity of examining different retinal vascular metrics in relation to multiple neurostructural metrics. Across all retinal measurements (AVR, CRAE, and CRVE), there was a significant negative relationship with cortical thickness, and significant positive associations with volume and surface area, in this region. The latter findings align with prior studies of adolescents with MetS, in whom retinal vascular caliber was positively associated with global measures gray matter volume and white matter integrity (Yau et al., 2014). Furthermore, a prior adult study found that CVRFs were significantly negatively associated with cortical thickness in the rostral middle frontal gyrus (Schwarz et al., 2018).

Overall, study findings reflect differences in the direction of vascular‐brain associations across different retinal vascular and neurostructural metrics. This variability may relate to several factors. During adolescence there are regional nonlinear decreases in brain volume and thickness with age, as well as complex regional and topographic patterns in the relationship between cortical thickness and surface area (Tamnes et al., 2017). With regard to cerebral vessels, there are known age and sex differences in regional CBF that may also influence cortical development (Satterthwaite et al., 2014). Furthermore, BD is associated with regional neurodevelopmental differences in comparison with HCs (Najt et al., 2016). Cortical metrics are strongly influenced by a number of genetic and cellular processes (Alexander‐Bloch et al., 2020; Geschwind & Rakic, 2013). Overall, there are multiple potential explanations for the variability in the direction of current findings. Ultimately, additional studies on this topic will be needed in order to generate both normative findings and disease‐specific findings.

### Diagnosis‐related findings

Vertex‐wise analyses detected one significant interaction effect, whereby greater AVR was associated with significantly smaller volumes in temporal and fusiform gyri in BD participants relative to HCs. It is unclear what the functional consequences of this relationship might be; while one may speculate that this is a deleterious association by virtue of the volumetric reduction, there are other examples of similar vascular‐brain associations that appear counter‐intuitive. For example, the SPRINT study found that better blood pressure control (i.e., lower blood pressure) was prospectively associated with greater *decreases* in total brain volume in adults (Nasrallah et al., 2019).

While we did observe a limited number of diagnosis‐specific interactions, it is interesting that the majority of our findings did not significantly differ between BD and HC adolescents. Psychotropic medication use may be contributory. While medications such as second‐generation antipsychotics, the most commonly used class of medications in the current sample, are clearly associated with traditional CVRFs, they do not appear to be meaningfully associated with CVD mortality (Osborn et al., 2015). This discordance may be due to the anti‐inflammatory and anti‐oxidative properties of most medications used in the treatment of BD (de Sousa et al., 2014; Gergerlioglu et al., 2007). Based on the existing evidence in adult BD and schizophrenia, it is likely that with increasing age and duration of illness, retinal microvascular differences will become more apparent between BD and HC participants (Appaji et al., 2019; Meier et al., 2013). Finally, while we found that the association between retinal vascular structure and brain structure did not significantly differ between adolescents with BD and HC, the clinical implications of this association may be accentuated in the BD group.

### Putative mechanisms

Owing to the absence of detailed literature examining vascular‐brain associations in youth, or psychiatric populations, it is unclear why the identified regions are more strongly associated with retinal microvascular caliber. There may be regional brain differences relating to cerebrovascular reactivity, microvascular supply, and cerebral metabolism. The lingual gyrus, which was associated with all retinal vascular metrics in this study, has been associated with reduced cerebrovascular reactivity in BD as compared to HC in a prior study from our group (Urback et al., 2019). Relatedly, CVRFs are associated with reduced lingual gyrus thickness in adults with type II diabetes mellitus (Yezhuvath et al., 2009).

In relation to arterial supply, anterior and posterior brain regions are supplied by different cerebral arteries, which potentially have different vulnerability to CVRFs (Hathaway & Newton, 2021; Jumah & Dossani, 2021; Spallazzi et al., 2019). We postulate that the link between retinal vascular caliber and brain structure may be mediated by differences in regional cerebral perfusion. Prior studies have found anomalous CBF to the ACC in BD (Toma et al., 2018). In other patient populations, including Alzheimer's disease, hypoperfusion is associated with reductions in brain volume (Benedictus et al., 2014); even subtle perfusion deficits during the key developmental epoch of adolescence may be consequential. Based on prior findings from our group, it appears that adolescents with BD have elevated resting cerebral perfusion (Toma, MacIntosh, et al., 2019), which may be related to known deficits in cerebral metabolism and mitochondrial function in BD (Andreazza et al., 2018). Relatedly, higher metabolism in those regions may be associated with greater inflammation and oxidative stress, which contribute to vascular endothelial dysfunction (Goldstein, 2017).

### Limitations

The results of this study should be interpreted in the context of several limitations. Although the sample size was relatively large for a single‐site neuroimaging study in adolescent BD, the study was underpowered for the interaction analyses. As detailed above, there is variability related to retinal vascular and neurostructural metrics that calls for meaningfully larger samples to address important secondary analyses. Second, this is a cross‐sectional study and therefore we cannot determine the direction of the association between microvascular caliber and brain structure. Third, signal detection may be more challenging compared to prior adult studies on this topic which primarily focus on brain diseases characterized by substantial neurostructural deficits (Heringa et al., 2013). Finally, variability related to scanner and to the lag between retinal imaging and brain scans may have diminished signal detection.

## CONCLUSION

This work addresses a significant gap in literature regarding the vascular‐brain interface in youth. Our findings suggest that optimizing cardiovascular health may offer direct brain benefits. Future research should focus on subpopulations where vascular‐brain associations may be particularly strong and for whom vascular‐targeted interventions may be most beneficial. Furthermore, while we chose to investigate vascular‐brain associations using two structural measures (retinal microvessel structure and brain structure), functional imaging measures may offer additional insights. Ultimately, this work is a first step that has generated preliminary insights for future studies and that is intended to encourage such future studies.

## CONFLICT OF INTERESTS

No conflicts declared.

## ETHIC STATEMENT

Consent was obtained from all participants and their parent and/or guardian prior to participating. Ethical approval was granted by Sunnybrook Research Institute Research Ethics Board. REB # 409/2013, 405/2014, 435/2015, 408/2011. All data was collected at Sunnybrook Research Institute. However, all data was transferred with the Centre for Youth Bipolar Disorder's relocation to the Centre for Addiction and Mental Health (CAMH). Thus, ethics approval was also granted by CAMH Research Ethics Board. REB # 152/2020, 163/2020, 165/2020, and 168/2020.

## AUTHOR CONTRIBUTIONS

Megan Mio primarily wrote the manuscript and performed statistical analyses. Kody G. Kennedy, Mikaela Dimick, and Alysha Sultan assisted in manuscript preparation and quality control of data. Dr. Benjamin I. Goldstein contributed to study conception and design and assisted with manuscript preparation. All authors contributed to revisions of the manuscript and have approved the final manuscript.

## Supporting information

Supporting Information S1

## Data Availability

The data that support the findings of this study are available on request from the corresponding author. The data are not publicly available due to privacy or ethical restrictions.
