# Supplementary material for: Neurostructural correlates of retinal microvascular caliber in adolescent bipolar disorder
Source: JCPP Adv. 2021 Aug 22;1(4):e12029. doi: 10.1002/jcv2.12029 (PMC10242848; doi:10.1002/jcv2.12029)
Supplement: Supplementary file 1 — Supporting Information S1 [file JCV2-1-e12029-s001.docx]

# Supporting Information

**Supplemental Methods** *1.1 Collection* *of Study Data* Study data were collected and managed using REDCap electronic data capture tools hosted at Sunnybrook Health Sciences Centre and later at the Centre for Addiction and Mental Health. REDCap (Research Electronic Data Capture) is a secure, web-based software platform designed to support data capture for research studies, providing 1) an intuitive interface for validated data capture; 2) audit trails for tracking data manipulation and export procedures; 3) automated export procedures for seamless data downloads to common statistical packages; and 4) procedures for data integration and interoperability with external sources (Harris et al., 2019; Harris et al., 2009).
*1.2 Diagnostic Assessment*
 The DSM-IV was published in 2013 and the DSM-5 version of K-SADS-PL was only available in 2016, therefore the current sample is based on DSM-IV criteria. Interviews were conducted by research staff with either a bachelor’s or master’s degree who received intensive training under the supervision of senior author B.I.G., a licensed child-adolescent psychiatrist. We opted to use the K-SADS-PL on participants ages 19-20 years in order to maintain comparability, given the small number of participants in that age group. Diagnoses of BD-I and BD-II were determined by DSM-IV criteria. BD-NOS was operationally defined using criteria published in the Course and Outcome of Bipolar Youth (COBY) study: i) significant change in functioning, ii) two DSM-IV symptoms of mania (three if only irritability is reported), iii) mood and symptom duration of at least 4 h during a 24 h period, and iv) at least 4 cumulative 24 h minimum episodes that meet mood, symptom, and functional change criteria over the participant’s lifetime.
*1.3 Assessment of Additional Demographic and Clinical Characteristics*
 Parental socioeconomic status was determined using the Hollingshead Four-Factor Index (Hollingshead, 1995). The Children’s Global Assessment Scale (CGAS) was used to collect global functioning during current and lifetime mood episodes (Shaffer et al., 1983). Family psychiatric history was collected via the Family History Screen (Weissman et al., 2000). The K-SADS-PL was used to assess lifetime tobacco smoking, which was computed as a “yes” or “no” variable. A Safety Form was used to record any lifetime police contact, physical and sexual abuse, as well as any suicidality or non-suicidal self-injury.

*1.4 Anthropometric Data*
 A Life Source Digital Blood Pressure Monitor was used to collect blood pressure. Measurement was repeated twice to ensure accuracy. BMI was calculated from height and weight measurements collected using standardized procedures (Krebs et al., 2007). To adjust for clothing differences, weight was deducted by 1.3kg for participants wearing long pants and long-sleeved shirts, 1.1kg for short pants or short-sleeved shirts, or 0.9kg for both short pants and short-sleeves.
*1.5 Retinal Photography*
 Diameters from the 3 largest arterioles and 3 largest venules were measured in accordance with a previously established protocol (Knudtson et al., 2003). Three individual raters measured each vessel at 3 equidistant points, after which the average of these measurements was taken for each vessel. Mean interclass correlation coefficient was 0.93 (CRAE: 0.90, CRVE: 0.95).
*1.6 Magnetic Resonance Imaging Acquisition and Processing*

Gray and white matter were quantified in 3D T1-weighted images, obtained using high-resolution fast-field echo imaging under the following parameters: i) 3T Philips Achieva System: TR/TE/TI= 9.5/2.3/1400ms, field of view 240 x 191mm^2^, spatial resolution 0.94x 1.17x 1.2 mm, 256x164x140 matrix, acquisition time (TA)= 8 minutes and 56 seconds, and ii) 3T Siemens Prisma System: TR/TE/TI= 2200/3.55/1090ms, isotropic 0.9x0.9x0.9mm voxel, acquisition time (TA)= 5 minutes and 7 seconds. Prior to preprocessing, images were assessed for motion and image artifacts by independent raters blinded to participant diagnosis. Each T1 image was given a score from 0-3 based on image quality, taking into account graininess, accurate segmentation between white and grey matter, and artifact due to participant movement in scanner. Steps including cortical reconstruction, parcellation, and sub-cortical segmentation of T1-weighted images were completed using the image analysis program FreeSurfer version 6.0 (http://surfer.nmr.mgh.harvard.edu/) (Dale, Fischl, & Sereno, 1999; Fischl et al., 2002; Fischl, Sereno, & Dale, 1999; Fischl, Sereno, Tootell, & Dale, 1999; Fischl et al., 2004; Ségonne et al., 2004; Sled, Zijdenbos, & Evans, 1998).
 Region of interest (ROI) volumes were created by adding individual gyral labels from the Desikan-Killiany atlas. The following ROIs were examined: i) anterior cingulate cortex (ACC), ii) ventrolateral prefrontal cortex (vlPFC) iii) ventromedial prefrontal cortex (vmPFC), iv) hippocampus, and v) amygdala. The ACC was composed of the caudal and rostral ACC, vlPFC included the pars triangularis and pars opercularis, and the vmPFC included the medial orbitofrontal. Amygdala and hippocampus were composed of their respective labels from left and right hemispheres. Mean thickness for each cortical ROI was calculated using the surface area of component regions as a weighting factor. For example, mean ACC thickness = [(cACC area x cACC thickness) + (rACC AREA x rACC thickness)] ﻿÷ (cACC + rACC area). Participants’ cortical thickness, volume, and surface area measures were exported from FreeSurfer. Regarding whole brain analysis, a 15mm kernel of full-width at half-maximum was used for smoothing. Volume, surface area, and cortical thickness data from each participant were mapped onto a canonical template.
*1.7 Statistical Analysis* Effect sizes were reported as Cohen’s *d* for continuous variables and Cramer’s *V* for categorical variables. Using G*Power 3.1 (Faul, Erdfelder, Lang, & Buchner, 2007), setting α=0.05, power=0.80, the sample size of 103 provides sufficient power to detect a small to medium effect size, *f^2^* ≥ 0.10 for ROI analyses. Owing to the absence of prior literature investigating retinal-brain associations within BD and the preliminary nature of this study, both uncorrected and corrected p-values are presented to inform future studies.

**References**

Dale, A. M., Fischl, B., & Sereno, M. I. (1999). Cortical surface-based analysis. I. Segmentation and surface reconstruction. *Neuroimage, 9*(2), 179-194. doi:10.1006/nimg.1998.0395

Faul, F., Erdfelder, E., Lang, A. G., & Buchner, A. (2007). G*Power 3: a flexible statistical power analysis program for the social, behavioral, and biomedical sciences. *Behav Res Methods, 39*(2), 175-191.

Fischl, B., Salat, D. H., Busa, E., Albert, M., Dieterich, M., Haselgrove, C., . . . Dale, A. M. (2002). Whole brain segmentation: automated labeling of neuroanatomical structures in the human brain. *Neuron, 33*(3), 341-355.

Fischl, B., Sereno, M. I., & Dale, A. M. (1999). Cortical surface-based analysis. II: Inflation, flattening, and a surface-based coordinate system. *Neuroimage, 9*(2), 195-207.

Fischl, B., Sereno, M. I., Tootell, R. B., & Dale, A. M. (1999). High-resolution intersubject averaging and a coordinate system for the cortical surface. *Hum Brain Mapp, 8*(4), 272-284.

Fischl, B., van der Kouwe, A., Destrieux, C., Halgren, E., Ségonne, F., Salat, D. H., . . . Dale, A. M. (2004). Automatically parcellating the human cerebral cortex. *Cereb Cortex, 14*(1), 11-22.

Harris, P. A., Taylor, R., Minor, B. L., Elliott, V., Fernandez, M., O'Neal, L., . . . Duda, S. N. (2019). The REDCap consortium: Building an international community of software platform partners. *J Biomed Inform, 95*, 103208.

Harris, P. A., Taylor, R., Thielke, R., Payne, J., Gonzalez, N., & Conde, J. G. (2009). Research electronic data capture (REDCap)--a metadata-driven methodology and workflow process for providing translational research informatics support. *J Biomed Inform, 42*(2), 377-381.

Hollingshead, P. A. (1995). Four-factor index of social status.

Knudtson, M. D., Lee, K. E., Hubbard, L. D., Wong, T. Y., Klein, R., & Klein, B. E. (2003). Revised formulas for summarizing retinal vessel diameters. *Curr Eye Res, 27*(3), 143-149.

Krebs, N. F., Himes, J. H., Jacobson, D., Nicklas, T. A., Guilday, P., & Styne, D. (2007). Assessment of child and adolescent overweight and obesity. *Pediatrics, 120 Suppl 4*, S193-228.

Ségonne, F., Dale, A. M., Busa, E., Glessner, M., Salat, D., Hahn, H. K., & Fischl, B. (2004). A hybrid approach to the skull stripping problem in MRI. *Neuroimage, 22*(3), 1060-1075.

Shaffer, D., Gould, M. S., Brasic, J., Ambrosini, P., Fisher, P., Bird, H., & Aluwahlia, S. (1983). A children's global assessment scale (CGAS). *Arch Gen Psychiatry, 40*(11), 1228-1231.

Sled, J. G., Zijdenbos, A. P., & Evans, A. C. (1998). A nonparametric method for automatic correction of intensity nonuniformity in MRI data. *IEEE Trans Med Imaging, 17*(1), 87-97.

Weissman, M. M., Wickramaratne, P., Adams, P., Wolk, S., Verdeli, H., & Olfson, M. (2000). Brief screening for family psychiatric history: the family history screen. *Arch Gen Psychiatry, 57*(7), 675-682.

**Supplemental Results**

|  | **ACC** | | **vlPFC** | | **vmPFC** | | **Amygdala** | | **Hippocampus** | |
| --- | --- | --- | --- | --- | --- | --- | --- | --- | --- | --- |
|  | **β** | **p** | **β** | **p** | **β** | **p** | **β** | **p** | **β** | **p** |
| *Volume* | | | | | | | | | | |
| AVR | 0.04 | 0.68 | 0.01 | 0.96 | -0.02 | 0.82 | 0.12 | 0.09 | 0.03 | 0.71 |
| CRAE | 0.20 | **0.049*** | -0.02 | 0.82 | 0.08 | 0.27 | 0.05 | 0.53 | -0.12 | 0.18 |
| CRVE | 0.12 | 0.21 | -0.04 | 0.67 | 0.09 | 0.21 | -0.07 | 0.35 | -0.15 | 0.08 |
| *Surface Area* | | | | | | | | | | |
| AVR | 0.05 | 0.63 | -0.03 | 0.78 | 0.02 | 0.74 |  | | | |
| CRAE | 0.19 | 0.06 | -0.04 | 0.71 | 0.10 | 0.19 |  |  |  |  |
| CRVE | 0.10 | 0.29 | -0.03 | 0.73 | 0.06 | 0.38 |  |  |  |  |
| *Cortical Thickness* | | | | | | | | | | |
| AVR | -0.07 | 0.49 | 0.01 | 0.92 | -0.06 | 0.53 |  | | | |
| CRAE | -0.13 | 0.18 | 0.00 | 0.99 | 0.07 | 0.54 |  |  |  |  |
| CRVE | -0.03 | 0.78 | -0.00 | 0.95 | 0.12 | 0.24 |  |  |  |  |

**Table S1. Retinal x diagnosis interaction** **effects on brain structure.**
ACC = anterior cingulate cortex; vlPFC = ventrolateral prefrontal cortex; vmPFC = ventromedial prefrontal cortex; AVR = arteriovenous ratio; CRAE = central retinal arteriolar equivalent; CRVE = central retinal venular equivalent. *** =** significant at *p*<0.05 before correction for multiple comparisons. **** =** significant at *p*<0.05 after correction for multiple comparisons.

**Supplemental Results Table S2.**

| **Retinal Measure** | **Peak Vertex Label** | **Encapsulated Region** | **Cortical Measure** | **Direction** | **Size of Cluster** | **cwp** | **MNI X** | **MNI Y** | **MNI Z** |
| --- | --- | --- | --- | --- | --- | --- | --- | --- | --- |
| **AVR** | Left isthmus cingulate cortex | Lingual gyrus, precuneus, cuneus, pericalcarine, lateral occipital cortex, superior parietal | Area | **+** | 7697.83 | <0.001 | -7.9 | -55.9 | 9.1 |
|  | Right isthmus cingulate cortex | Precuneus, cuneus, lingual gyrus, pericalcarine, lateral occipital cortex, fusiform gyrus | Area | **+** | 8594.16 | <0.001 | 17.7 | -50.9 | 5.8 |
|  | Right rostral middle frontal gyrus | Pars orbitalis, pars triangularis, lateral orbitofrontal cortex, frontal pole | Area | **+** | 3567.81 | 0.0015 | 23.8 | 43.6 | 20.6 |
|  | Left rostral middle frontal gyrus | Pars orbitalis, lateral orbitofrontal cortex, frontal pole | Thickness | **-** | 1443.41 | 0.03 | -14.3 | 58.0 | -15.8 |
|  | Left precuneus | Lingual gyrus, cuneus, fusiform gyrus, isthmus cingulate cortex, posterior cingulate, pericalcarine, lateral occipital cortex, superior parietal lobe | Volume | **+** | 9250.18 | <0.001 | -8.6 | -69.9 | 37.0 |
|  | Right isthmus cingulate cortex | Precuneus, cuneus, lateral occipital cortex, lingual gyrus, fusiform gyrus, pericalcarine, | Volume | **+** | 8580.62 | <0.001 | 20.8 | -52.7 | 6.5 |
|  | Right rostral middle frontal gyrus | Pars triangularis, superior frontal gyrus | Volume | **+** | 2019.43 | 0.006 | 39.4 | 46.5 | 3.1 |
| **CRAE** | Left isthmus cingulate cortex | Precuneus, cuneus, pericalcarine, lingual gyrus, lateral occipital cortex | Area | **+** | 6684.82 | <0.001 | -9.0 | -55.7 | 9.3 |
|  | Right rostral middle frontal gyrus | Pars orbitalis, pars triangularis, lateral orbitofrontal cortex | Area | **+** | 2979.86 | 0.006 | 39.4 | 50.6 | -2.9 |
|  | Right lateral occipital cortex | Cuneus, fusiform gyrus, superior parietal lobe | Area | **+** | 4866.33 | <0.001 | 22.9 | -90.2 | 18.8 |
|  | Left supramarginal gyrus | Superior temporal and transverse temporal gyri, insula | Thickness | **-** | 1750.29 | 0.008 | -49.7 | -29.3 | 17.8 |
|  | Left rostral middle frontal gyrus | Lateral orbitofrontal cortex, pars orbitalis | Thickness | **-** | 1659.32 | 0.014 | -14.1 | 56.3 | -16.4 |
|  | Left precuneus | Cuneus, pericalcarine, lingual gyrus, isthmus cingulate cortex, superior parietal lobe, lateral occipital cortex | Volume | **+** | 6347.52 | <0.001 | -8.8 | -69.6 | 36.6 |
|  | Right lateral occipital cortex | Fusiform gyrus, cuneus, pericalcarine | Volume | **+** | 5592.44 | <0.001 | 28.9 | -94.6 | -9.6 |
|  | Right isthmus cingulate cortex | Precuneus, lingual gyrus, pericalcarine, parahippocampal gyrus | Volume | **+** | 1784.87 | 0.012 | 20.0 | -52.3 | 6.4 |
| **CRVE** | Left precuneus | Cuneus, pericalcarine, lingual gyrus, isthmus cingulate cortex, lateral occipital cortex, superior parietal lobe | Area | **+** | 7649.61 | <0.001 | -8.5 | -56.8 | 9.6 |
|  | Right lateral occipital cortex | Fusiform gyrus, cuneus, superior parietal lobe | Area | **+** | 8284.71 | <0.001 | 14.1 | -50.0 | 5.3 |
|  | Right rostral middle frontal gyrus | Pars orbitalis, pars triangularis, lateral orbitofrontal cortex | Area | **+** | 3506.43 | 0.0016 | 23.6 | 43.3 | 21.3 |
|  | Left precuneus | Cuneus, pericalcarine, lingual, isthmus cingulate cortex, posterior cingulate, lateral occipital cortex, superior parietal lobe | Volume | **+** | 7301.29 | <0.001 | -8.6 | -69.9 | 37.0 |
|  | Right lateral occipital cortex | Fusiform gyrus, cuneus, pericalcarine, superior parietal lobe | Volume | **+** | 6328.84 | <0.001 | 29.3 | -94.4 | -10.2 |
|  | Right isthmus cingulate cortex | Precuneus, lingual gyrus, pericalcarine, parahippocampal gyrus | Volume | **+** | 2134.85 | 0.004 | 19.8 | -52.6 | 6.7 |
|  | Right rostral middle frontal gyrus | Pars triangularis, superior frontal | Volume | **+** | 1744.12 | 0.013 | 22.4 | 33.1 | 32.8 |
|  | Left rostral middle frontal gyrus | Lateral orbitofrontal cortex, pars orbitalis, frontal pole | Thickness | **-** | 1882.74 | 0.004 | -13.3 | 56.7 | -16.9 |
|  | Left supramarginal gyrus | Transverse temporal and superior temporal gyri, insula | Thickness | **-** | 1834.07 | 0.005 | -48.7 | -30.1 | 17.9 |

**Table S2. Significant retinal main effect clusters detected via vertex-wise analysis.**AVR = arteriovenous ratio, CRAE = central retinal arteriolar equivalent, CRVE = central retinal venular equivalent.

**Supplemental Results Table S3.**

| **Retinal Measure** | **Peak Vertex Label** | **Encapsulated Region** | **Cortical Measure** | **Direction** | **Size of Cluster** | **p-value** | **MNI X** | **MNI Y** | **MNI Z** |
| --- | --- | --- | --- | --- | --- | --- | --- | --- | --- |
| **AVR** | Left middle temporal | Inferior temporal, superior temporal, and fusiform gyri | Area | **-** | 2979.34 | 0.004 | -61.2 | -13.7 | -21.1 |

**Table S3. Significant retinal x diagnosis interaction effect cluster detected via vertex-wise analysis.**AVR = arteriovenous ratio
